# MESPool: Molecular Edge Shrinkage Pooling for hierarchical molecular representation learning and property prediction

**DOI:** 10.1093/bib/bbad423

**Published:** 2023-12-02

**Authors:** Fanding Xu, Zhiwei Yang, Lizhuo Wang, Deyu Meng, Jiangang Long

**Affiliations:** School of Life Science and Technology, Xi’an Jiaotong University, 710049 Shaanxi, China; School of Physics, Xi’an Jiaotong University, 710049 Shaanxi, China; School of Life Science and Technology, Xi’an Jiaotong University, 710049 Shaanxi, China; Rearch Institute for Mathematics and Mathematical Technology, Xi’an Jiaotong University, 710049 Shaanxi, China; School of Mathematics and Statistics, Henan University, 475004 Henan, China; School of Life Science and Technology, Xi’an Jiaotong University, 710049 Shaanxi, China

**Keywords:** GNN, graph pooling, functional group, molecular property prediction

## Abstract

Identifying task-relevant structures is important for molecular property prediction. In a graph neural network (GNN), graph pooling can group nodes and hierarchically represent the molecular graph. However, previous pooling methods either drop out node information or lose the connection of the original graph; therefore, it is difficult to identify continuous subtructures. Importantly, they lacked interpretability on molecular graphs. To this end, we proposed a novel Molecular Edge Shrinkage Pooling (MESPool) method, which is based on edges (or chemical bonds). MESPool preserves crucial edges and shrinks others inside the functional groups and is able to search for key structures without breaking the original connection. We compared MESPool with various well-known pooling methods on different benchmarks and showed that MESPool outperforms the previous methods. Furthermore, we explained the rationality of MESPool on some datasets, including a COVID-19 drug dataset.

## INTRODUCTION

Molecular property prediction is a fundamental task in drug discovery, and plays a crucial role in computer-aided drug discovery workflows [1] because many methods rely on predicted molecular properties to evaluate, select and generate molecules [2]. In recent years, AI-driven molecular property prediction methods have become a hot spot in the field of lead compound discovery and optimization [1, 3]. At the same time, graph neural networks (GNNs) have shown their power in graph representation learning [4, 5], and have been further applied to molecular graph data [6, 7]. Traditional machine learning methods require hand-crafted molecular features, such as molecular fingerprints and descriptors [8, 9]. By comparison, GNNs learn high-dimensional embeddings of atoms and bonds through message passing (or graph convolution) end to end and therefore represent the graph structures and interactions of molecules.

A general message passing scheme smooths node signals across the graph by an aggregation operation and implicitly learns the structural information. However, it is difficult to distinguish task-related structures and unrelated parts [10], or hierarchically represent the graph. To address these problems and obtain refined graph local representations, local pooling layers can be inserted into typical GNNs, similar to convolutional neural networks (CNNs) [11]. Graph local pooling operations hierarchically reduce the graph representation and preserve the local structural information of interest.

Previous graph pooling methods can be grossly classified into two categories: sparse pooling [12–14] and dense pooling [15–18], according to their node selection method. Liu *et al*. [19] listed and summarized twenty representative graph pooling methods. The characteristics of sparse pooling and dense pooling can be summarized as follows:

- Sparse pooling:
  (i) Sparse pooling aims to preserve the task-related nodes and drop out the unrelated nodes in each layer.
  (ii) Some structural information will be lost in the process of node dropping, but the original connection will be preserved.
  (iii) A threshold (minimum score) can be set to adaptively adjust the number of pooled nodes.
  (iv) The number of parameters will not increase with the increasing graph size.
- Dense pooling:
  (i) Dense pooling aims to cluster nodes and hierarchically represent the graph in each layer.
  (ii) A fixed ratio or number of pooling clusters is always needed.
  (iii) Dense pooling does not drop the node information, but the rebuilt connection of the pooled graph is not strongly related to the original graph.
  (iv) The number of parameters is related to the size of the graph. Thus dense pooling requires much more computational resources than sparse pooling on large graphs.

Specifically, most pooling methods are hardly interpretable, yet interpretability is critical in drug- and medical-related tasks [19].

To this end, we proposed a novel graph pooling method called Molecular Edge Shrinkage Pooling (MESPool). MESPool is inspired by the concept of molecular scaffold’ and functional group’, and MESPool aims to gradually shrink the scaffold and functional groups into supernodes in a similar way to the scaffold tree [20]. With the deepening of the network, the crucial edges are preserved, and the graph is simplified into the connection of functional groups. Different from the previous methods, we regard edges (chemical bonds) as the basic pooling unit instead of nodes (atoms). MESPool scores edges with their features and uses a threshold to adaptively adjust the pooling proportion. As a consequence, we also proposed a convolution operator edge-featured graph isomorphism network (EGIN), which introduced edge feature updates based on a graph isomorphism network (GIN) [21].

MESPool has the following advantages:

(i) It has the ability to distinguish the task-related structures and hierarchically represent the graph.
(ii) It maintains the original graph connection without missing any node information.
(iii) The pooled graph contains original nodes and supernodes, where supernodes represent disjoint substructures.
(iv) The number of parameters is fixed and will not increase with the increasing of the graph size.
(v) More importantly, the pooling result of MESPool can provide good rationality and further serves as a valuable guide for interpretability, as discussed in the Results.

**Table 1:** Common notations used throughout this paper

Notation	Description
$\mathcal{G}$	A mathematical graph.
$\mathcal{V}$	Set of nodes.
$\mathcal{E}$	Set of edges.
$\mathcal{N}(i)$	The neighbor node set of node $i$.
$n$	The number of nodes.
$m$	The number of edges.
$d_{v}$	The dimension of a node feature vector.
$d_{e}$	The dimension of an edge feature vector.
$\boldsymbol{h}_{i} \in \mathbb{R}^{d_{v}}$	Feature vector of node $i$.
$\boldsymbol{h}_{i \to j} \in \mathbb{R}^{d_{e}}$	Feature vector of directed edge $i \to j$.
$\boldsymbol{h}_{e(i,j)} \in \mathbb{R}^{d_{e}}$	Feature vector of undirected edge $e(i,j)$.
$\boldsymbol{H}_{\mathcal{V}} \in \mathbb{R}^{n \times d_{v}}$	Feature matrix of all nodes in $\mathcal{V}$.
$\boldsymbol{H}_{\mathcal{E}} \in \mathbb{R}^{m \times d_{e}}$	Feature matrix of all nodes in $\mathcal{E}$.
$\boldsymbol{A}\in{\left \{0,1\right \}}^{n\times n}$	Adjacency matrix.
$\boldsymbol{W}$	Weight matrix of a linear layer.
$b$	Bias of a linear layer.
$\sigma $	A non-linear activation function.
$\operatorname{MLP}$	Multilayer Perceptron.
$\operatorname{BN}$	Batch normalization.

**Table 2:** SRC functions of the baseline pooling method

Methods	Selection	Reduction	Connection
**TopK**	$\boldsymbol{y}=\frac{\boldsymbol{H}_{\mathcal{V}} \boldsymbol{p}}{\|p\|}$ ; $\boldsymbol{i}=\operatorname{TOP}_{k}(\boldsymbol{y})$.	$\boldsymbol{H}_{\mathcal{V}}^{\prime }=\left (\boldsymbol{H}_{\mathcal{V}} \odot \sigma (\boldsymbol{y})\right )_{\boldsymbol{i}}$	$\boldsymbol{A}^{\prime }=\boldsymbol{A}_{\boldsymbol{i}, \boldsymbol{i}}$
**SAGPool**	$\boldsymbol{y}=\operatorname{GNN}\left (\boldsymbol{A}, \boldsymbol{H}_{\mathcal{V}}\right )$ ; $ \boldsymbol{i}=\mathrm{TOP}_{k}(\boldsymbol{y})$	$\boldsymbol{H}_{\mathcal{V}}^{\prime }=\left (\boldsymbol{H}_{\mathcal{V}} \odot \sigma (\boldsymbol{y})\right )_{\boldsymbol{i}}$	$\boldsymbol{A}^{\prime }=\boldsymbol{A}_{\boldsymbol{i}, \boldsymbol{i}}$
**DiffPool**	$\boldsymbol{S}=\operatorname{GNN}_{1}\left (\boldsymbol{A}, \boldsymbol{H}_{\mathcal{V}}\right )$	$\boldsymbol{H}_{\mathcal{V}}^{\prime }=\boldsymbol{S}^{\top } \operatorname{GNN}_{2}\left (\boldsymbol{A}, \boldsymbol{H}_{\mathcal{V}}\right )$	$\boldsymbol{A}^{\prime }=\boldsymbol{S}^{\top } \boldsymbol{A} \boldsymbol{S}$
**MinCut**	$\boldsymbol{S}=\operatorname{MLP}\left (\boldsymbol{H}_{\mathcal{V}}\right )$	$\boldsymbol{H}_{\mathcal{V}}^{\prime }=\boldsymbol{S}^{\top }\boldsymbol{H}_{\mathcal{V}}$	$\boldsymbol{A}^{\prime }=\boldsymbol{S}^{\top } \boldsymbol{A} \boldsymbol{S}$
**ASAP**	$\boldsymbol{S}=\operatorname{Master2Token}\left (\boldsymbol{H}_{\mathcal{V}}\right )$	$\boldsymbol{H}_{\mathcal{V}}^{c}=\boldsymbol{S}^{\top } \boldsymbol{H}_{\mathcal{V}}$ ; $\boldsymbol{\phi }=\operatorname{LEConv}\left (\boldsymbol{H}_{\mathcal{V}}^{c}\right )$; $\hat{\boldsymbol{H}}_{\mathcal{V}}^{c}=\boldsymbol{\phi } \odot \boldsymbol{H}_{\mathcal{V}}^{c}$; $\boldsymbol{i}=\operatorname{TOP}_{k}\left (\hat{\boldsymbol{H}}_{\mathcal{V}}^{c}\right )$; $\hat{\boldsymbol{S}}=(\boldsymbol{S})_{\boldsymbol{i}}$; $\boldsymbol{H}_{\mathcal{V}}^{\prime }=\left (\hat{\boldsymbol{H}}_{\mathcal{V}}^{c}\right )_{\boldsymbol{i}}$.	$\hat{\boldsymbol{A}}=\boldsymbol{A}+\boldsymbol{I}$ ; $\boldsymbol{A}^{\prime }=\hat{\boldsymbol{S}}^{\top }\hat{\boldsymbol{A}}\hat{\boldsymbol{S}}$.
**EdgePool**	$r_{i \rightarrow j}=\boldsymbol{W}\left (\boldsymbol{h}_{i} \| \boldsymbol{h}_{j}\right )+b$ ; $s_{i \rightarrow j}=0.5+\operatorname{softmax}_{\mathcal{N}(j)}\left (r_{i \rightarrow j}\right )$	$\boldsymbol{h}_{\textrm{super} (i j)}=s_{i \rightarrow j}\left (\boldsymbol{h}_{i}+\boldsymbol{h}_{j}\right )$	Edges are reorganizedafter iteration.
MESPool*	$s_{e(i, j)}=\sigma \left (\boldsymbol{s}^{\top } \boldsymbol{h}_{e(i, j)}\right )$ ; $\mathcal{E}_{\textrm{pool}}=\left \{e(i, j) \mid s_{e(i, j)}<\textrm{threshold}\right \}$; $\mathcal{V}_{\textrm{pool}}=\left \{i, j \mid s_{e(i, j)}<\textrm{threshold}\right \}$	$\left \{\mathcal{V}_{\mathrm{subset}\left (1\right )},\ldots ,\mathcal{V}_{\mathrm{subset}\left (K\right )}\right \} $ $=\operatorname{CONNECTED}(\mathcal{V}_{\mathrm{pool}},\mathcal{E}_{\mathrm{pool}})$; $\boldsymbol{h}_{\mathrm{super}(k)}=\sum _{i\in \mathcal{V}_{\mathrm{subset}(k)}}\boldsymbol{h}_{i}^{\prime }$	$\mathcal{V}_{\mathrm{new}}=(\mathcal{V}-\mathcal{V}_{\mathrm{pool}})\cup \mathcal{V}_{\mathrm{super}}$ ; $\mathcal{E}_{\mathrm{new}}=\operatorname{RECONNECT}\left (\mathcal{E}-\mathcal{E}_{\mathrm{pool}}\right )$

*See Methods for details of MESPool framework.

**Figure 1:**
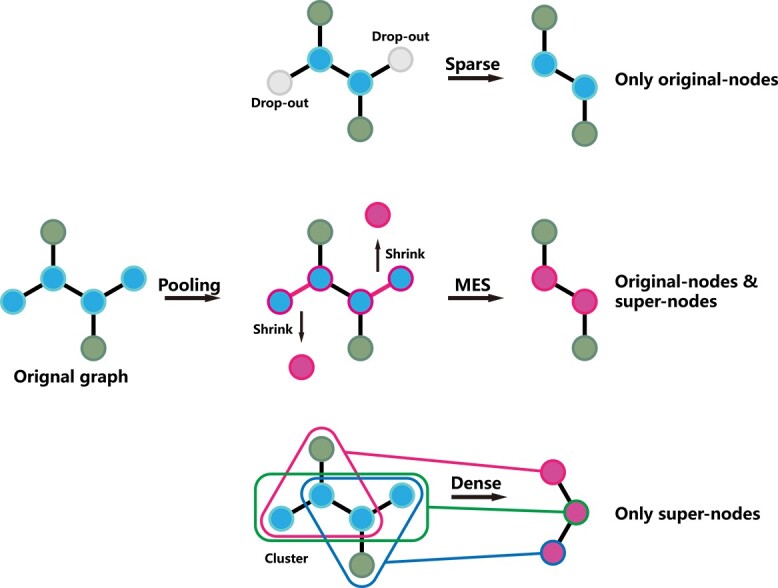
Diagram of the pooling process: sparse pooling drops out nodes and obtains node information missing from the original graph; dense pooling groups nodes into supernodes (an original node will be contained in different supernodes at once) and obtains a brand new graph; MESPool shrinks part of the disjoint structures into supernodes and keeps the original connection.

## RELATED WORKS

### Graph neural networks

A graph can be represented as $\mathcal{G}=(\mathcal{V},\mathcal{E})$, where the node set $\mathcal{V}$ contains $\lvert \mathcal{V}\rvert =n$ nodes and the edge set $\mathcal{E}$ contains $\lvert \mathcal{E}\rvert =m$ edges. In addition, an adjacency matrix $\boldsymbol{A}\in{\left \{0,1\right \}}^{n\times n}$ is normally used to describe the connections in a graph (We listed the common notations and their descriptions in Table 1). The main idea of GNNs is to learn node feature representation by iteratively aggregating node features from neighbors and integrating the aggregated information with the central node representation. A general GNN scheme (or a graph convolution layer) can be formalized as a message passing (MP) function:


(1) \begin{align*}& \begin{split} \boldsymbol{h}_{i}^{(l)}=\operatorname{UPDATE}^{(l)}\left(\boldsymbol{h}_{i}^{(l-1)}, \operatorname{AGGREGATE}^{(l)} \right.\\ \left. \left(\left\{\boldsymbol{h}_{j}^{(l-1)}, \forall i \in \mathcal{N}(j)\right\}\right)\right) \end{split}\end{align*}

where $\boldsymbol{h}_{i}^{(l)}\in \mathbb{R}^{d_{v}}$ is the representation of node $i$ at the $l$th GNN layer. $\operatorname{AGGREGATE}^{(l)}$ and $\operatorname{UPDATE}^{(l)}$ denote the functions of the aggregation operation and update operation at the $l^{\mathrm{th}}$ GNN layer, respectively. Finally, an entire graph representation $\boldsymbol{h}_{\mathcal{G}}^{(l)}\in \mathbb{R}^{d}$ can be obtained by a readout (global pooling) function: 


(2) \begin{align*}& \boldsymbol{h}_{\mathcal{G}}^{(l)}=\operatorname{READOUT}^{(l)}\left(\left\{\boldsymbol{h}_{i}^{(l)} \mid i \in \mathcal{V}\right\}\right)\end{align*}

Various GNNs have been proposed in recent years. Kipf and Welling [22] proposed the graph convolution network (GCN), which simplified the approximation of the graph Laplacian using the Chebyshev expansion method [23]. GraphSAGE [24] learns node embeddings through sampling and aggregation. graph attention network (GAT) [25] introduced attention mechanisms to calculate the weight of nodes while propagating. In addition, Xu *et al*. [21] discussed the design principles of aggregation and update operations and proposed the graph isomorphism network (GIN).

### Graph pooling

Graph local pooling (hereinafter referred to as graph pooling) layers allow GNNs to obtain graph local structural information hierarchically by reducing the number of nodes. Grattarola *et al*. [26] summarized the graph pooling operator as the combination of three functions: selection, reduction and connection (SRC). The selection function groups nodes into subsets (dense pooling) or just selects the important nodes (sparse pooling); then, the reduction function aggregates subsets into supernodes or just deletes the uninterested nodes; finally, the connection function relinks the reduced nodes and outputs a pooled graph.

Sparse pooling exploits learnable scoring functions to delete nodes with lower significance scores. As a representative sparse pooling method, TopK [12, 13] scores nodes based on a learnable projection vector, keeps high-scoring nodes and drops out low-scoring nodes. SAGPool [14] improves TopK by using a GNN to score nodes to consider both node features and graph topology.

Dense pooling considers graph pooling as a node clustering problem, and it groups nodes into a fixed number of clusters by computing a cluster assignment matrix. DiffPool [15] and MinCut [16] use GNN and MLP to compute the cluster assignment matrix, respectively, and constrain the rationality of clustering with regularization terms.

In addition, there are some other interesting pooling methods. ASAP [27] is a mixed method that introduces a self-attention mechanism Master2Token into the general dense pooling process to consider the information inside the clusters. Furthermore, ASAP scores clusters and drops out the low-scoring clusters. Unlike the other methods, EdgePool [28, 29] is a distinctive hard pooling method; it scores edges, traverses the graph and contracts half of the edges that are high-scoring but nonadjacent.

The SRC functions of the above methods are listed in Table 2 as baseline methods. Besides these classic methods, some new methods also presented intriguing ideas. HGP-SL [30] is a sparse pooling method, which selects nodes that are more representative of their neighbors by calculate the Manhattan distance between nodes. Haar graph pooling [31] applies the Haar basis system to compress the graph, it generates groupings of nodes following a series of clustering methods. TAP [32] selects important nodes by a two-stage voting (local and global) process to consider the topology of the graph. However, these methods still follow a comparable framework to the sparse and dense pooling methods commonly used.

**Table 3:** Initial featurization

Feature	Description	Length
**Atom features**		120
**Atom type**	Type of atom (atomic number one-hot encoding).	100
**Aromaticity**	The atom is or is not part of an aromatic system.	1
**Hs**	Number of bonded hydrogen atoms.	5
**Formal charge**	The electronic charge assigned to the atom.	5
**Chirality**	The chirality type of the atom.	4
**Hybridization**	The hybridization form or the atom.	5
**Bond features**		13
**Bond type**	Type of bond.	5
**Conjugation**	The bond is or is not conjugated.	1
**In ring**	The bond is or is not part of a ring.	1
**Stereo**	The stereo type of the bond.	6

**Figure 2:**
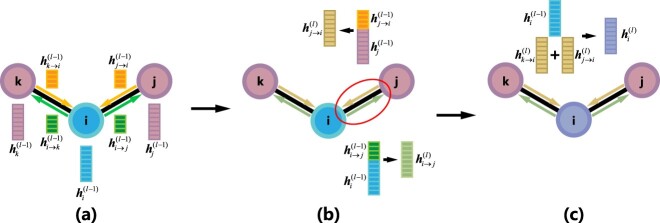
The illustration of EGIN process. (**A**) The features of nodes and edges (initially, $\boldsymbol{h}_{i\rightarrow j}^{(0)}=\boldsymbol{h}_{j\rightarrow i}^{(0)}=\boldsymbol{h}_{e(i,j)}^{(0)}$); (**B**) an example of edge updating (Edge $i\rightarrow j$ and $j\rightarrow i$ update with their starting node $i$ and $j$) (undirected edges convert into directed edges); (**C**) a central node ($i$) update with its neighbor edges ($j\rightarrow i$ and $k\rightarrow i$).

A diagrammatic sketch of the comparison between MESPool and sparse/dense pooling is shown in Figure 1. The core idea of MESPool is to discriminate the edges within functional groups and the edges connecting the functional groups, and abstract the molecular graph into the connection of functional group supernodes. MESPool divides the graph in a way conforming to chemical intuition and preserves the original connection relationship of the graph.

## METHODS

### Edge-featured Graph Isomorphism Network

Edges with their features play an essential role in many real-world graph data. For molecular graphs, edge features describe chemical bond type, conjugation, ring and stereo information (the initial atom and bond features are listed in Table 3). In addition, an edge can represent the smallest substructure in the graph with two connected nodes, and we call this unit the smallest pooling substructure in our MESPool. To update and propagate the edge features, we propose the EGIN, which alternately updates edge features and node features based on the framework of the GIN.

**Figure 3:**
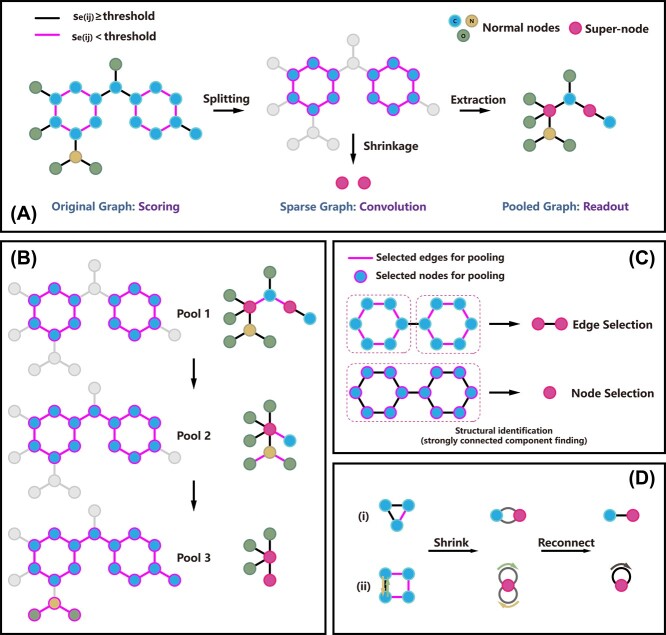
(**A**) The pooling process of a MESPool layer; (**B**) the concept illustration of a three-layer MESPool network; (**C**) the difference between edge selection and node selection: In our architecture, the selected edges/nodes will be clustered and pooled into supernodes according to their connection. Edge selection can distinguish the connected substructures better in this case; (**D**) special cases of shrinkage, repeated edges will be added into one edge: (i) a ring preserving two adjacent edges will be converted into two nodes connected with two edges after shrinkage; (ii) a ring preserving one edge will be converted into a supernode with two self-loops after shrinkage, since the original preserved edge is directed.

The nodewise formulation of the GIN can be described as [21] 


(3) \begin{align*}& \boldsymbol{h}_{i}^{(l)}=\operatorname{MLP}\left((1+\epsilon) \cdot \boldsymbol{h}_{i}^{(l-1)}+\sum_{j \in \mathcal{N}(i)} \boldsymbol{h}_{j}^{(l-1)}\right)\end{align*}

where $\epsilon $ is a learnable parameter that adjusts the weight of the central node. The adjacent node messages are aggregated through a summation function, and the central node feature is then updated by a multilayer perceptrons.

In an EGIN layer, the edge feature will be updated first by concatenating its starting node feature, and undirected edges will be converted to directed edges through this operator (Figure 2A,B): 


(4) \begin{align*}& \boldsymbol{h}_{i\rightarrow j}^{\left(l\right)}=\sigma(\boldsymbol{W}(\boldsymbol{h}_{i\rightarrow j}^{\left(l-1\right)}\|\boldsymbol{h}_{i}^{\left(l-1\right)})+b)\end{align*}

where $\boldsymbol{h}_{i\rightarrow j}^{\left (l\right )} \in \mathbb{R}^{d_{e}}$ denotes the feature of directed edge $i\rightarrow j$ at the $l$th layer, $\boldsymbol{W}$ is a learnable transformation matrix and $b$ is a learnable bias. Since the updated edge feature $\boldsymbol{h}_{i\rightarrow j}^{\left (l\right )}$ contains the information of directed edge $i\rightarrow j$ and the starting node $i$, we can treat $\boldsymbol{h}_{i\rightarrow j}^{\left (l\right )}$ as the weighted message of neighbor node $i$ to central node $j$. Therefore, the aggregation function becomes the summation of neighboring edge features, and the node update operator can be written as follows: 


(5) \begin{align*}& \boldsymbol{h}_{i}^{(l)}=\operatorname{MLP}\left(\boldsymbol{h}_{i}^{(l-1)} \| \sum_{k \in \mathcal{N}(i)} \boldsymbol{h}_{k \rightarrow i}^{(l)}\right)\end{align*}

We use the concatenate function to combine the central node feature $\boldsymbol{h}_{i}^{\left (l-1\right )}$ and the aggregated message $\sum _{k\in \mathcal{N}(i) \backslash j}\boldsymbol{h}_{k\rightarrow i}^{\left (l\right )}$ because they are different kinds of features and may have the different dimensions.

**Figure 4:**
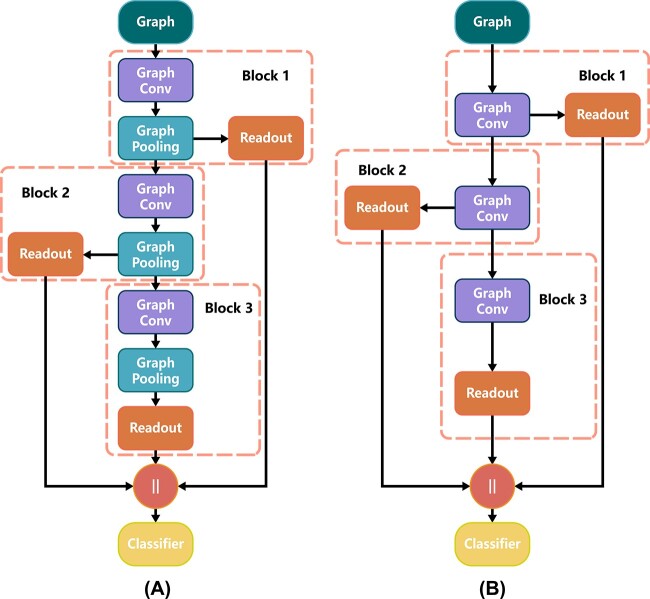
Model architecture with pooling layers (**A**) and without pooling layers (**B**).

**Table 4:** Classification benchmark results

Dataset	BACE	BBBP	MUV	HIV	SIDER	Tox21	Clintox
**Molecules**	1513	2039	93 087	41 127	1402	7815	1476
**Tasks**	1	1	17	1	27	12	2
**Positive ratio**	0.457	0.765	0.002	0.035	0.571	0.078	0.506
**Metric**	AUC-ROC (Higher is better)						
**GIN**	0.824 +/− 0.055	0.788 +/− 0.075	0.702 +/− 0.032	0.722 +/− 0.036	0.568 +/− 0.019	0.755 +/− 0.020	0.885 +/− 0.076
**EGIN**	0.810 +/− 0.079	0.784 +/− 0.109	0.701 +/− 0.041	0.717 +/− 0.040	0.574 +/− 0.023	0.774 +/− 0.017	0.836 +/− 0.194
**TopKPool (GIN)**	0.813 +/− 0.056	0.801 +/− 0.070	0.710 +/− 0.040	0.738 +/− 0.026	0.561 +/− 0.028	0.771 +/− 0.021	0.853 +/− 0.100
**SAGPool (GIN)**	0.790 +/− 0.060	0.768 +/− 0.079	0.684 +/− 0.056	0.721 +/− 0.024	0.534 +/− 0.029	0.737 +/− 0.032	0.817 +/− 0.133
**DiffPool (GIN)**	0.817 +/− 0.058	0.802 +/− 0.053	0.704 +/− 0.062	0.704 +/− 0.037	0.560 +/− 0.028	0.770 +/− 0.026	0.901 +/− 0.070
**MinCut (GIN)**	0.814 +/− 0.054	0.809 +/− 0.063	0.710 +/− 0.038	0.738 +/− 0.023	–	–	0.897 +/− 0.072
**ASAP (GIN)**	0.797 +/− 0.060	0.773 +/− 0.077	0.684 +/− 0.063	0.720 +/− 0.038	0.566 +/− 0.021	0.746 +/− 0.033	0.771 +/− 0.162
**EdgePool (GIN)**	0.838 +/− 0.055	0.787 +/− 0.053	0.698 +/− 0.039	0.721 +/− 0.028	0.566 +/− 0.017	0.759 +/− 0.017	0.881 +/− 0.068
**TopKPool (EGIN)**	0.810 +/− 0.054	0.799 +/− 0.082	0.696 +/− 0.036	0.742 +/− 0.024	0.555 +/− 0.027	0.776 +/− 0.021	0.879 +/− 0.090
**SAGPool (EGIN)**	0.819 +/− 0.044	0.819 +/− 0.061	0.708 +/− 0.056	0.740 +/− 0.025	0.565 +/− 0.031	0.784 +/− 0.019	0.873 +/− 0.097
**EdgePool (EGIN)**	0.838 +/− 0.050	0.791 +/− 0.063	0.727 +/− 0.035	0.732 +/− 0.037	0.570 +/− 0.016	0.778 +/− 0.022	0.830 +/− 0.090
**MESPool (EGIN)**	**0.855 +/− 0.039**	**0.848 +/− 0.046**	**0.754 +/− 0.030**	**0.756 +/− 0.032**	**0.576 +/− 0.026**	**0.787 +/− 0.019**	**0.902 +/− 0.065**
**Metric**	AUC-PRC (Higher is better)						
**GIN**	0.813 +/− 0.062	0.909 +/− 0.052	0.045 +/− 0.022	0.280 +/− 0.051	0.609 +/− 0.039	0.367 +/− 0.041	0.799 +/− 0.087
**EGIN**	0.803 +/− 0.071	0.912 +/− 0.064	0.054 +/− 0.026	0.241 +/− 0.059	0.607 +/− 0.046	**0.414 +/− 0.038**	0.739 +/− 0.139
**TopKPool (GIN)**	0.806 +/− 0.068	0.916 +/− 0.048	0.024 +/− 0.016	0.252 +/− 0.046	0.608 +/− 0.055	0.362 +/− 0.039	0.710 +/− 0.136
**SAGPool (GIN)**	0.797 +/− 0.063	0.900 +/− 0.051	0.022 +/− 0.027	0.230 +/− 0.050	0.592 +/− 0.060	0.315 +/− 0.052	0.698 +/− 0.126
**DiffPool (GIN)**	0.794 +/− 0.073	0.918 +/− 0.043	0.034 +/− 0.026	0.217 +/− 0.054	0.604 +/− 0.051	0.370 +/− 0.048	0.825 +/− 0.086
**MinCut (GIN)**	0.793 +/− 0.075	0.921 +/− 0.046	0.035 +/− 0.020	0.261 +/− 0.042	–	–	0.803 +/− 0.103
**ASAP (GIN)**	0.798 +/− 0.066	0.902 +/− 0.050	0.033 +/− 0.027	0.253 +/− 0.053	0.608 +/− 0.042	0.361 +/− 0.051	0.686 +/− 0.138
**EdgePool (GIN)**	0.838 +/− 0.071	0.907 +/− 0.043	0.040 +/− 0.021	0.272 +/− 0.042	0.605 +/− 0.042	0.380 +/− 0.037	0.796 +/− 0.079
**TopKPool (EGIN)**	0.797 +/− 0.067	0.915 +/− 0.052	0.023 +/− 0.021	0.258 +/− 0.045	0.605 +/− 0.047	0.360 +/− 0.040	0.727 +/− 0.129
**SAGPool (EGIN)**	0.807 +/− 0.055	0.927 +/− 0.041	0.029 +/− 0.027	0.261 +/− 0.044	0.611 +/− 0.053	0.381 +/− 0.041	0.736 +/− 0.125
**EdgePool (EGIN)**	0.831 +/− 0.063	0.910 +/− 0.045	0.056 +/− 0.035	0.283 +/− 0.038	0.608 +/− 0.039	0.411 +/− 0.045	0.736 +/− 0.072
**MESPool (EGIN)**	**0.849 +/− 0.052**	**0.940 +/− 0.033**	**0.079 +/− 0.044**	**0.298 +/− 0.035**	**0.612 +/− 0.040**	**0.391 +/− 0.033**	**0.811 +/− 0.078**

Note: **Bold** denotes the highest mean value; underline denotes the lowest standard deviation, ‘-’ means failed to obtain a valid result.

**Table 5:** Regression benchmark results

Dataset	ESOL	FreeSolv	Lipophilicity
**Molecules**	1128	639	4200
**Tasks**	1	1	1
**Metric**	RMSE (lower is better)		
**GIN**	1.446 +/− 0.256	3.757 +/− 0.898	0.805 +/− 0.039
**EGIN**	**1.130 +/− 0.141**	2.822 +/− 0.863	0.753 +/− 0.044
**TopKPool (GIN)**	1.507 +/− 0.253	3.458 +/− 0.989	0.950 +/− 0.103
**SAGPool (GIN)**	1.452 +/− 0.222	4.035 +/− 0.897	0.962 +/− 0.110
**DiffPool (GIN)**	1.792 +/− 0.678	3.589 +/− 0.912	0.975 +/− 0.112
**MinCut (GIN)**	1.254 +/− 0.264	3.431 +/− 0.881	0.813 +/− 0.046
**ASAP (GIN)**	1.541 +/− 0.676	4.014 +/− 0.986	0.969 +/− 0.112
**EdgePool (GIN)**	1.187 +/− 0.167	2.993 +/− 0.827	0.807 +/− 0.049
**TopKPool (EGIN)**	1.601 +/− 0.333	3.757 +/− 1.159	0.916 +/− 0.087
**SAGPool (EGIN)**	1.622 +/− 0.409	3.627 +/− 1.129	0.913 +/− 0.100
**EdgePool (EGIN)**	1.235 +/− 0.295	3.086 +/− 0.940	0.752 +/− 0.042
**MESPool (EGIN)**	1.276 +/− 0.246	**2.779 +/− 0.762**	**0.708 +/− 0.042**

Note: **Bold** denotes the lowest mean value; underline denotes the lowest standard deviation.

In the following sections, we simply express the graphwise EGIN process as follows: 


(6) \begin{align*}& \boldsymbol{H}_{\mathcal{E}}^{(l)}, \boldsymbol{H}_{\mathcal{V}}^{(l)}=\operatorname{EGIN}\left(\boldsymbol{H}_{\mathcal{E}}^{(l-1)}, \boldsymbol{H}_{\mathcal{V}}^{(l-1)}\right)\end{align*}

### Molecular Edge Shrinkage Pooling

In this section, we describe the components of our MESPool following the SRC scheme. The main idea of MESPool is to preserve crucial edges and shrink other structures into supernodes (see Figure 3A, B). Unlike the previous methods, MESPool initially selects edges (units) to pool rather than nodes. Edge selection can distinguish the connected substructures better than node selection in this framework(see Figure 3C), which is helpful for hierarchical representation. MESPool can be seen as the mixture of sparse and dense pooling, which select and split the units by scoring like sparse pooling, instead of dropping out low scored nodes (units), MESPool reduces their clusters into supernodes like dense pooling. In such a process, MESPool not only maintains the connection of the original graph, but also does not lose any node information. In addition, since the scaffolds are shrunk into nodes, the distance between side chains is shortened on the pooled graph, as the combination and interaction of functional groups can be represented in the deeper network after pooling.

#### Selection: threshold splitting

In the selection process, we score the undirected edges to represent the weight of the corresponding units, and the adjacent low-scored units will be considered as a subset (pooling substructure). Therefore, it is necessary to consider the adjacency information when scoring an edge. Initially, we adopt an edge message propagation in the selection process: 


(7) \begin{align*}& \begin{aligned} \boldsymbol{h}_{i \rightarrow j}^{\mathrm{p}}&=\operatorname{MLP}\left(\boldsymbol{h}_{i \rightarrow j}+\sum_{k \in \mathcal{N}(i)} \boldsymbol{h}_{k \rightarrow i}\right) \\ \boldsymbol{h}_{e(i, j)}&=\boldsymbol{h}_{i \rightarrow j}^{\mathrm{p}}+\boldsymbol{h}_{j \rightarrow i}^{\mathrm{p}} \end{aligned}\end{align*}

Note that the directed edge features here have contained the information of the starting nodes, thanks to the edge update operator in the EGIN layer before the pooling layer. Consequently, the undirected edge feature $\boldsymbol{h}_{e(i,j)}$ can represent the information of the corresponding unit and its neighboring units.

The scoring function is a linear transformation on $\boldsymbol{h}_{e(i,j)}$: 


(8) \begin{align*}& s_{e(i, j)}=\sigma\left(\boldsymbol{s}^{\top} \boldsymbol{h}_{e(i, j)}\right)\end{align*}

where $s_{e(i,j)}$ is the score of undirected edge $e(i,j)$, $\boldsymbol{s}\in \mathbb{R}^{d_{e}}$ is a learnable scoring operator and $\sigma $ denotes the sigmoid function. The edge features can be further updated by the score: 


(9) \begin{align*}& \begin{aligned} \boldsymbol{h}_{i \rightarrow j}^{\prime} &=s_{e(i, j)} \cdot \boldsymbol{h}_{i \rightarrow j}^{\mathrm{p}} \\ \boldsymbol{h}_{j \rightarrow i}^{\prime} &=s_{e(i, j)} \cdot \boldsymbol{h}_{j \rightarrow i}^{\mathrm{p}} \end{aligned}\end{align*}

According to the edge score, units can be divided into two groups by a manually set hyperparameter $\lambda $. In addition, the average value of edge scores $s_{\mathrm{mean}}$ is used to control the number of pooling units and ensure that the layer will not pool all the nodes at once. Consequently, we have a threshold that can be denoted as the minimum value of $\lambda $ and $s_{\mathrm{mean}}$: 


(10) \begin{align*}& \mathrm{threshold} = \operatorname{MIN}(\lambda, s_{\mathrm{mean}})\end{align*}

The pooling edge and node set can be further described as follows: 


(11) \begin{align*}& \begin{gathered} \mathcal{E}_{\mathrm{pool}} =\left\{e(i, j) \mid s_{e(i, j)}<\mathrm{threshold}, \forall i,j \right\}\\ \mathcal{V}_{\mathrm{pool}}=\left\{i, j \mid s_{e(i, j)}<\mathrm{threshold}, \forall i,j \right\} \end{gathered}\end{align*}

**Figure 5:**
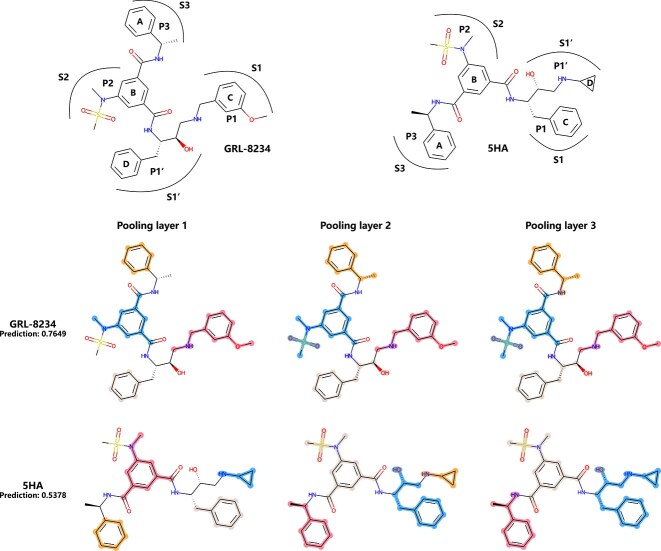
Pooling visualization results of the BACE1 inhibitors GRL-8234 and 5HA (highlight colors are only used to distinguish supernodes, not representing any properties).

**Figure 6:**
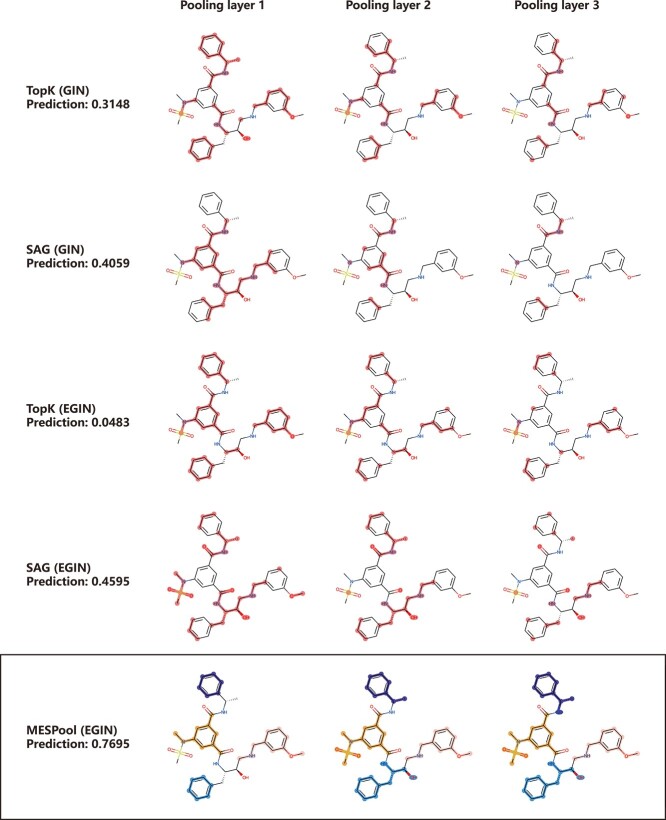
Comparison of MESPool and sparse pooling methods for visualization results of GRL-8234

**Figure 7:**
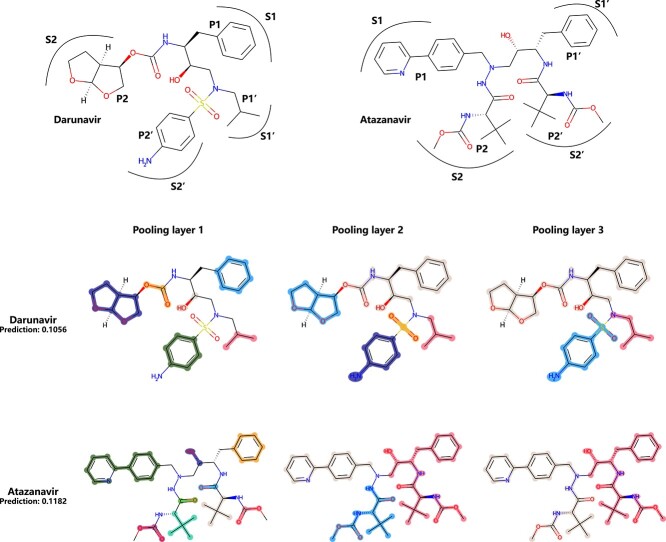
Pooling visualization results of the HIV inhibitors Darunavir and Atazanavir (highlight colors are only used to distinguish supernodes, not representing any properties).

**Figure 8:**
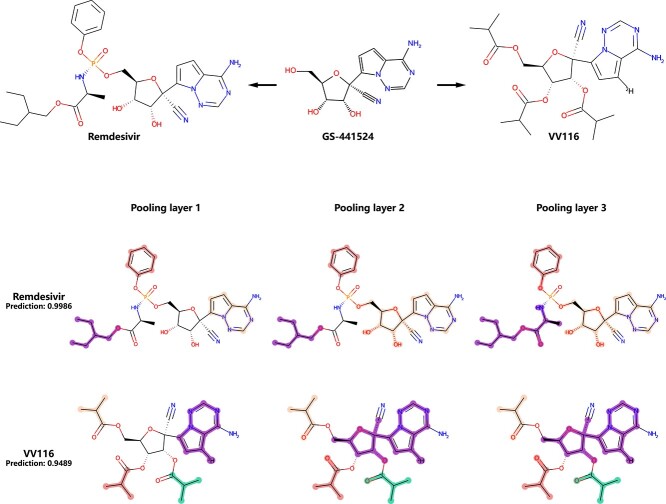
Original structure of the COVID-19 drugs Remdesivir, VV116 and GS-441524; and the pooling visualization of Remdesivir and VV116 (highlight colors are only used to distinguish supernodes, not representing any properties).

#### Reduction: unit shrinkage

After the pooling set is split out, a strongly connected component finding algorithm [33], $\operatorname{CONNECTED}$, is used to group the connected pooled nodes into subsets: 


(12) \begin{align*}& \left\{\mathcal{V}_{\mathrm{subset}\left(1\right)},\ldots,\mathcal{V}_{\mathrm{subset}\left(K\right)}\right\}=\operatorname{CONNECTED}(\mathcal{V}_{\mathrm{pool}},\mathcal{E}_{\mathrm{pool}})\end{align*}

where $\mathcal{V}_{\mathrm{subset}\left (\cdot \right )}$ is a node set representing an independent pooled substructure, $K$ is the calculated number of subsets in the layer, and we have 


(13) \begin{align*}& \mathcal{V}_{\mathrm{subset}\left(i\right)}\cap\mathcal{V}_{\mathrm{subset}\left(j\right)}=\emptyset,\ \ \forall i,j\in{1,2,\ldots,K}\end{align*}

The pooled edge and node set $\mathcal{E}_{\mathrm{pool}}$ and $\mathcal{V}_{\mathrm{pool}}$ represents a sparse graph with multiple discrete subgraphs $\left \{\mathcal{V}_{\mathrm{subset}\left (1\right )},\ldots , \mathcal{V}_{\mathrm{subset}\left (K\right )}\right \}$; therefore, a GNN can be used to learn the representations of subgraphs. Here, we apply the EGIN on $\mathcal{E}_{\mathrm{pool}}$ and $\mathcal{V}_{\mathrm{pool}}$: 


(14) \begin{align*}& \boldsymbol{H}_{\mathcal{E}_{\mathrm{pool}}}^{\prime},\boldsymbol{H}_{\mathcal{V}_{\mathrm{pool}}}^{\prime}=\operatorname{EGIN}\left(\boldsymbol{H}_{\mathcal{E}_{\mathrm{pool}}},\boldsymbol{H}_{\mathcal{V}_{\mathrm{pool}}}\right)\end{align*}

A normal node featured GNN can also be usable here, since only the output node features will be used in the subsequent process. The subsets can be further shrunk into supernodes with the updated node features: 


(15) \begin{align*} & \boldsymbol{h}_{\mathrm{super}(k)}=\sum_{i\in\mathcal{V}_{\mathrm{subset}(k)}}\boldsymbol{h}_{i}^{\prime}\end{align*}

(16) \begin{align*} & \mathcal{V}_{\mathrm{super}}=\{\mathrm{super}(k) \mid k \in\{1,2, \ldots, K\}\}\end{align*}

where $\boldsymbol{h}_{i}^{\prime }$ is the $i$th row of $\boldsymbol{H}_{\mathcal{V}_{\mathrm{pool}}}^{\prime }$ and $\mathrm{super}(k)$ denotes the $k^{\mathrm{th}}$ supernode. Since pooled substructures are shrunk into supernodes, their influence on the whole graph will be reduced in the deeper network, and we consider the pooled part to be task unrelated.

#### Connection: preserved connection optimization

After reduction, the node set of the new graph can be described as follows: 


(17) \begin{align*}& \mathcal{V}_{\mathrm{new}}=(\mathcal{V}-\mathcal{V}_{\mathrm{pool}})\cup\mathcal{V}_{\mathrm{super}}\end{align*}

In several special cases, there will be some node pairs in $\mathcal{V}_{new}$ connected with multiple edges or one node with multiple self-loops. Therefore, we optimize the connection by summing the repeated edges that link the same nodes into one edge (see Figure 3D): 


(18) \begin{align*}& \mathcal{E}_{\mathrm{new}}, \boldsymbol{H}_{\mathcal{E}_{\mathrm{new}}}=\operatorname{RECONNECT}\left(\mathcal{E}-\mathcal{E}_{\mathrm{pool}}, \boldsymbol{H}_{\mathcal{E}-\mathcal{E}_{\mathrm{pool}}}\right)\end{align*}

Eventually, the new graph after pooling can be denoted as follows: 


(19) \begin{align*}& \mathcal{G}_{\mathrm{new}}=(\mathcal{V}_{\mathrm{new}},\mathcal{E}_{\mathrm{new}})\end{align*}

## RESULTS

### Experiment

We evaluate the proposed MESPool and previous pooling methods on molecular classification and regression tasks. In this section, we describe the model architecture, benchmark datasets and training strategy and finally summarize the results of our experiments.

#### Model architecture

In the experiment, we adopt a unified model architecture [14] (Figure 4A) for all baseline pooling methods (Table 2) and MESPool to perform a horizontal comparison. The architecture contains three blocks, and each block consists of a graph convolution layer and a graph pooling layer. Since the previous pooling methods are node-based, we use GIN as the default convolution layer. Additionally, we add a control group with EGIN for TopK, SAGPool and EdgePool as a supplement, since they are able to process edge features. We use EGIN as the convolution layer of MESPool because MESPool relies on edge features. We apply a summation readout function at the end of each block, aggregating the node features to obtain the hierarchical graph representation: 


(20) \begin{align*}& \boldsymbol{h}_{\mathcal{G}}=\sum_{i \in \mathcal{V}} \boldsymbol{h}_{i}\end{align*}

Finally, the output hierarchical graph representations are concatenated and passed to a linear layer for classification. In addition, a pure GNN architecture without hierarchical pooling layers is also used to test the GIN and EGIN as a comparison (Figure 4B).

#### Datasets

Seven classification and three regression benchmark datasets are selected in our experiment. **BACE** [34] is a dataset of molecules that provides quantitative IC50 and qualitative (binary label) binding results for a set of inhibitors of human beta-secretase 1 (BACE-1). **BBBP** [35] includes binary labels for over 2000 compounds on their permeability properties. The **HIV** dataset contains over 40 000 compounds with binary labels representing the ability to inhibit HIV replication. **MUV** [36] is a benchmark dataset containing 17 challenging tasks for approximately 90 000 compounds that were selected from PubChem BioAssay. **SIDER** [37, 38] groups drug side effects into 27 system organ classes and contains over 1400 approved drugs. The **Tox21** dataset contains over 8000 compounds and their qualitative toxicity measurements on 12 different targets. **ClinTox** [39, 40] compares drugs approved by the FDA and drugs that have failed clinical trials for toxicity reasons, encompassing two classification tasks and over 1400 drugs. The **ESOL** (Delaney) [41] dataset is a regression dataset containing structures and water solubility data for 1128 compounds. The **FreeSolv** [42] dataset is a collection of experimental and calculated hydration free energies for small molecules in water, along with their experimental values. The **Lipophilicity** dataset curated from ChEMBL database, provides experimental results of octanol/water distribution coefficient (logD at pH 7.4) of 4200 compounds.

#### Training strategy

The initial atom and bond features are listed in Table 3, and all the methods adopt the same initial featurization. In the experiment, we adopt the random scaffold splitting [43, 44] procedure to split the dataset (training:validation:test = 8:1:1), which splits the molecules according to their scaffold, making the prediction more challenging than random splitting. We take 5 random seeds to split each dataset and apply 10 independent runs for each split. A total of 50 testing results were used to report the mean and standard deviation of the performance. In fairness to all pooling methods, we adopt the same early stop criterion, patience and Adam optimizer. The relevant values were set to be suitable and tolerant for all methods based on pre-experiment. The hyperparameters (including hidden dimensions, pooling ratio, dropout ratio and learning rate) are independently tuned for each method by grid searching.

#### Benchmark results

Table 4 shows the performance of MESPool on seven classification tasks in comparison to baseline models. The results are evaluated by the area under receiver operating characteristic curve (AUC-ROC) and the area under precision-recall curve (AUC-PRC). Table 5 is the benchmark results on three regression tasks evaluated by the root mean squared error (RMSE). Overall, MESPool outperforms other pooling methods on most datasets. Most baseline pooling methods have no significant performance improvement compared with the GIN network without pooling layers, especially BACE, MUV and HIV.

Additionally, comparing the models with GIN and EGIN, the performance of SAGPool improved on both seven datasets when using EGIN, while the performance of TokPool and EdgePool had no significant change. In addition, introducing the edge features did not make the performance of EGIN ahead of GIN; in contrast, EGIN has a very poor performance on Clintox. By comparison, MESPool can always maintain a decent performance. This shows that the advantage of MESPool is not due to directly introducing the edge feature but to the special design of the pooling layer.

### Rationality analysis

In this section, we choose two benchmark datasets (BACE and HIV) and a novel dataset of potential drugs against SARS-CoV-2 and give some examples to analyze the rationality of MESPool. We use some reported drugs to predict their properties, visualize their pooling results and compare them with their structure design principle. Additionally, we give some pooling examples on two commonly used tasks (ESOL and Mutagenicity) to explain the rationality on functional group identification.

Here, we take two molecules for each dataset to describe in this section. In general, the first pooling layer tends to identify and shrink the ring structure. With the deepening of the network, more core regions are found, especially the recognition of connection structures such as acetamide. Finally, the molecule is divided into several key functional groups, and the chemical bonds between them are retained (details below).

To approach the actual application scenario, the benchmark datasets are split by random splitting here (training: validation: test = 8:1:1) and retrained. The AUC-ROC results of BACE and HIV are $0.922+/-0.024$ and $0.813+/-0.016$, respectively, after 10 independent runs.

#### BACE

$\beta $ -site amyloid precursor protein cleaving enzyme 1 (BACE1) is the $\beta $-secretase enzyme required for the production of the neurotoxic $\beta $-amyloid (A$\beta $) peptide. The inhibition of BACE1 is one of the important therapeutic approaches of Alzheimer’s disease [45, 46]. The binary classification label of the BACE dataset is qualitative from the $\mathrm{qIC}_{50}$ results. Inhibitors are labeled as 1, and noninihibitors are labeled as 0. Figure 5 shows the pooling results of two compounds, GRL-8234 ($\mathrm{IC}_{50}=1~\mathrm{nM}$) [45, 47] and 5HA ($\mathrm{IC}_{50}=15~\mathrm{nM}$) [45, 48, 49]. GRL showed excellent selectivity for BACE-1; however, 5HA showed poor BBB penetration.

The two molecules have a common isophthalamide scaffold (structure B). GRL-8234 has a 3-methoxybenzyl group at P1 (which is critical for the enhanced cellular inhibitory properties) and a phenylalanine side chain at P1’. On the other hand, 5HA has a cyclopropyl moiety oriented toward the S1’ subpocket of the enzyme active site. The pooling visualization results show that the first layer identified four key ring structures, and in the deeper layers, supernodes expand from the rings. Finally, the functional groups that make hydrophobic contacts in BACE-1 binding pockets are recognized and shrunken.

Significantly, the BACE dataset has strict requirements for $\mathrm{qIC}_{50}$ values, it is challenging to accurately predict GRL-8234 and 5HA. We specifically focus on GRL-8234 as a representative example (Figure 6), visualized the pooling results of TopK and SAG, to demonstrate the superior rationality of our method. The baseline methods both mistakenly predicted the drug GRL-8234 with a score below 0.5. As the network deepens, the loss of structural information becomes increasingly pronounced, leaving behind sparse structures that lack chemical rationality. In contrast, we firmly believe that the accuracy of our predictions stems from the rationality of our structural selection, which is the very aspect distinguishes our method from others.

#### Human immunodeficiency virus

Human immunodeficiency virus (HIV) is one of the main causes of morbidity and mortality worldwide [50]. The HIV dataset collects the information on the ability of compounds to inhibit HIV replication, and the labels show the HIV activity: confirmed active and moderately active are labeled 1, and confirmed inactive is labeled 0. Highly active antiretroviral therapy (HAART) is recognized as the most effective treatment method for AIDS, and protease inhibitors play a very important role in HAART [51]. Here, we selected two protease inhibitors approved by the FDA, Darunavir [51, 52] and Atazanavir [51, 53], to discuss the rationality of the pooling results (Figure 7).

Darunavir has a similar structure to amprenavir; they both have a benzyl group at the P1 site, and an isobutyl group at P1’ connects the phenyl amide P2’ group by a sulfonamide. The main design of Darunavir is a bicyclic tetrahydrofuran (bis-THF) at the P2 site, which can effectively hydrogen bond with both Asp-29 and Asp-30 NHs present in the S2 subsite [52]. These feature functional groups are basically identified during the pooling process. Atazanavir exhibits potent anti-HIV activity, and a unique structural characteristic is the presence of a large phenylpyridyl P1 group that is asymmetric relative to its benzyl P1’ group. The symmetrical and asymmetrical structures of atazanavir are found at the beginning of pooling. Overall, the compounds are finally divided from the vicinity of acetylamine, which is a similar binding pattern of FDA-approved HIV protease inhibitors [51].

#### COVID dataset

The Coronavirus disease 2019 (COVID-19) pandemic caused by Severe Acute Respiratory Syndrome Coronavirus-2 (SARS-CoV-2) has spread worldwide since it was first identified in December 2019 [54, 55]. It has become a very popular and important research direction to uncover and develop antiviral interventions in recent years [56]. We made a COVID-19 (anti-SARS-CoV-2) dataset that combined the dataset used by Wang *et al*. [57] and potential drugs in DrugDevCovid19 (http://clab.labshare.cn/covid/php/index.php), including 1140 positive compounds and 1862 negative compounds (total 3002 compounds). The dataset is split via random splitting (training:validation:test=8:1:1), and the AUC-ROC after 10 independent runs is $0.936+/-0.006$. We select two COVID-19 drugs, Remdesivir [56] and VV116 [58], to discuss here (Figure 8).

Remdesivir and VV116 are both RNA-dependent RNA polymerase (RdRp) inhibitors, and they are designed on the nucleoside analog core GS-441524. GS-441524 is a prodrug that is able to diffuse into cells and slowly convert into nucleoside monophosphate via phosphorylation and further processed into an active nucleoside triphosphate derivative with phosphokinase to inhibit RdRp. Remdesivir is the monophosphate of GS-441524, and the additional functional groups accelerate the phosphorylation process [56]. On the other hand, the tri-isobutyrate ester VV116 obtained good oral bioavailability through the esterification of 7-deuterated GS-441524 [58]. We can see from Figure 8 (C) that the pooling layers identified the nucleoside analog core on Remdesivir and VV116. Furthermore, the monophosphate structure is preserved on remdesivir, and the added benzyl and polar/nonpolar mixed functional group are shrunk respectively. In addition, three isobutyryl groups on VV116 were also identified and shrunk.

**Figure 9:**
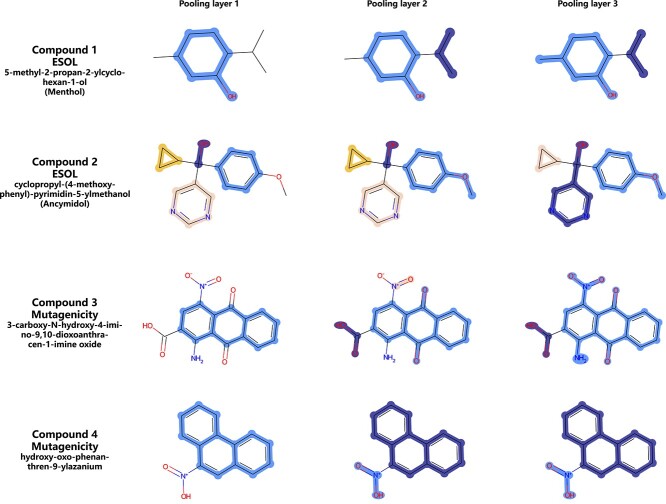
Pooling visualization results of four instance compounds on two tasks, ESOL and Mutagenicity (highlight colors are only used to distinguish supernodes, not representing any properties).

#### Functional group indetification

Water solubility and mutagenicity are both important and widely studied tasks. Wu *et al*. [59] proposed a GNN based structure-activity relationship (SAR) mining method named substructure mask explanation (SME), which is based on well-established molecular segmentation methods (BRICS substructures [60], Murcko substructures [61] and manually set functional groups). It analyses task-related structures through the voting of consensus model. Our pooling method shows similar results to these molecular segmentation methods.

The RMSE result of ESOL with random splitting after 10 independent runs is $0.748 +/- 0.022$. The Mutagenicity dataset in [59] contains 7672 compounds and 1 binary label, and the AUC-ROC result is $0.913 +/- 0.008$. Figure 9 shows the pooling results of 4 instance compounds on the two tasks. The results indicate a pattern that closely resembles BRICS substructure segmentation, particularly for compound 1 and 3. For the solubility task, MESPool identifies multiple functional groups that are consistent with the known chemical knowledge, including hydroxyl, pyrimidine, isopropyl and cyclopropane. In the mutagenicity prediction task, toxicophores such as aromatic nitro, aromatic amine, quinones and polycyclic aromatic system are combined, and separate from the detoxifying group carboxylic acid.

## CONCLUSION

We proposed a novel graph pooling method for molecule representation learning called MESPool, which reduces structures by selecting edges and is able to adaptively adjust the pooling ratio. The biggest difference from previous methods is that MESPool can search task-relevant structures directly on the original graph, which makes MESPool rational. In this study, we train the three-block network for molecular property prediction end-to-end without any prior information. MESPool shows better performance than the baseline methods. Meanwhile, the pooling results have shown a good chemical intuition and are consistent with the drug design logic.

Learning to find the key substructures is an important subject in many molecular-related tasks. In addition to property prediction, there are also drug–drug interactions [62], drug–protein interactions [63] and molecule generation [64]. We are looking forward to applying the idea of MESPool to more research fields. We believe it is also an interesting study direction in the future to strengthen and stabilize the interpretability of MESPool by introducing chemical prior information through pretraining and other methods, and further enable the identification of novel functional groups.

**Key Points**

- We proposed a novel edge-based graph pooling method called MESPool, it shows better performance than previous methods on molecular property prediction tasks.
- MESPool identifies task-relevant substructures in a graph, making it rational for molecular representation learning.
- Comparing to previous methods, MESPool maintains the original graph connection without missing any node information.
- We introduced edge feature updating based on the framework of the GIN, that alternately updates edge features and node features.

## Data Availability

The benchmark datasets of this work can be found at https://deepchemdata.s3-us-west-1.amazonaws.com/datasets/BBBP.csv (replace the BBBP in the hyperlink with another dataset name to download other datasets). The COVID-19 dataset was collected from http://clab.labshare.cn/covid/php/index.php and https://github.com/pkuwangsw/COVIDVS [57].

## CODE AVAILABILITY

The code of this work is open sourced and available at https://github.com/xfd997700/MESPool.

## Notes

**Fanding Xu** received the B.E. degree in biomedical engineering from Taiyuan University of Technology, China, in 2019. He is currently pursuing the Ph.D. degree in biomedical engineering at Xi’an Jiaotong University. His current research interest is in the area of AI driven drug discovery, especially the development and application of graph neural networks and geometric deep learning.

**Zhiwei Yang** is presently serving as an Associate Professor of School of Physics at Xi’an Jiaotong University. He got the Ph.D. degree in Northeast Forestry University in 2011, and then worked as postdoc in Xi’an Jiaotong University. He started his independent research in 2016. He has 14 years of experience in the research of dynamic structure, functional mechanism and artificial regulation of biological macromolecules. He has published over 100 peer-reviewed papers in SCI-indexed journals and 3 chapters of academic monographs.

**Lizhuo Wang** received the Bachelor’s and Master’s degrees in Electrical Engineering from Texas A&M University at College Station, TX, USA. Currently pursuing a Ph.D. in Biology at Xi’an Jiaotong University, his research interest is AI for science, particularly in addressing biological challenges through computational approaches.

**Deyu Meng** (Member, IEEE) received the B.Sc., M.Sc., and Ph.D. degrees in Applied Mathematics from Xi’an Jiaotong University, Xi’an, China, in 2001, 2004, and 2008, respectively. From 2012 to 2014, he took his two-year sabbatical leave with Carnegie Mellon University, Pittsburgh, PA, USA. He is a Professor with the School of Mathematics and Statistics, Xi’an Jiaotong University. His research interests include model-based deep learning, variational networks, and meta-learning.

**Jiangang Long** recived the B.S. degree in Pharmacy, M.S. degree in Public Health and Ph.D. degree in Biochemistry and Molecular Biology from Second Military Medical University and Chinese Academy of Sciences, in 1996, 2002 and 2006 respectively. He worked as Postdoctoral fellow in University of California, CA, USA (2006–2008), and Banner (Sun) Health Research Institute, AZ, USA (2008–2009). He is currently a Biology Professor with the School of Life Science and Technology, and Vice Dean of Graduate School at Xi’an Jiaotong University. His research interests include Mitochondrial Degeneration & Therapeutic Intervention in Neurodegenerative Diseases (AD) and Mitochondrial Dysfunction Related Diseases.

## APPENDIX

### Convolution layer selection

Our study aims to compare various pooling layers horizontally, and as such, we have opted for a compact and representative convolution layer. GIN stands out for its exceptional discriminative power, thanks to its association with the Weisfeiler–Lehman test. It has been proven to possess high representational power both theoretically and empirically. Above all, GIN’s simple summation aggregation has minimal impact on substructure identification, unlike the attention mechanism in GAT and the sampling process in GraphSAGE. As a result, the pooling layer assumes a leading role in structural identification within the GIN framework. The benchmark results for GCN, GAT, GIN and GraphSAGE are presented in Tables 6 and 7, none of these methods exhibit a significant advantage over the others in the majority of tasks.

**Table 6:** Classification benchmark results of convolution networks

Dataset	BACE	BBBP	MUV	HIV	SIDER	Tox21	Clintox
**Molecules**	1513	2039	93 087	41 127	1402	7815	1476
**Tasks**	1	1	17	1	27	12	2
**Positive ratio**	0.457	0.765	0.002	0.035	0.571	0.078	0.506
**Metric**	AUC-ROC (higher is better)						
**GCN**	0.772 +/− 0.075	0.770 +/− 0.084	0.592 +/− 0.048	0.690 +/− 0.037	0.521 +/− 0.027	0.602 +/− 0.051	0.847 +/− 0.070
**GAT**	0.797 +/− 0.060	**0.804 +/− 0.059**	0.669 +/− 0.042	0.730 +/− 0.026	0.569 +/− 0.022	0.765 +/− 0.018	**0.888 +/− 0.086**
**GIN**	**0.824 +/− 0.055**	0.788 +/− 0.075	0.702 +/− 0.032	0.722 +/− 0.036	0.568 +/− 0.019	0.755 +/− 0.020	0.885 +/− 0.076
**GraphSAGE**	0.811 +/− 0.047	0.804 +/− 0.073	**0.742 +/− 0.025**	**0.748 +/− 0.031**	**0.574 +/− 0.022**	**0.778 +/− 0.019**	0.796 +/− 0.189
**Metric**	AUC-PRC (Higher is better)						
**GCN**	0.733 +/− 0.054	0.895 +/− 0.058	0.234 +/− 0.147	0.197 +/− 0.074	**0.635 +/− 0.069**	0.235 +/− 0.121	0.659 +/− 0.088
**GAT**	0.779 +/− 0.052	**0.919 +/− 0.043**	0.020 +/− 0.016	0.245 +/− 0.040	0.607 +/− 0.044	0.378 +/− 0.045	0.754 +/− 0.128
**GIN**	**0.813 +/− 0.062**	0.909 +/− 0.052	**0.045 +/− 0.022**	0.280 +/− 0.051	0.609 +/− 0.039	0.367 +/− 0.041	0.799 +/− 0.087
**GraphSAGE**	0.793 +/− 0.052	0.914 +/− 0.052	0.037 +/− 0.024	**0.285 +/− 0.043**	0.608 +/− 0.045	**0.404 +/− 0.040**	0.666 +/− 0.107

Note: **Bold** denotes the highest mean value; underline denotes the lowest standard deviation.

**Table 7:** Regression benchmark results of convolution networks

Dataset	ESOL	FreeSolv	Lipophilicity
**Molecules**	1128	639	4200
**Tasks**	1	1	1
**Metric**	RMSE (Lower is better)		
**GCN**	1.718 +/− 0.491	4.281 +/− 1.093	0.852 +/− 0.035
**GAT**	**1.246 +/− 0.304**	**3.491 +/− 0.963**	0.829 +/− 0.057
**GIN**	1.446 +/− 0.256	3.757 +/− 0.898	**0.805 +/− 0.039**
**GraphSAGE**	1.340 +/− 0.245	3.910 +/− 0.870	0.833 +/− 0.050

Note: **Bold** denotes the lowest mean value; underline denotes the lowest standard deviation.

**Table 8:** Classification benchmark results of dense pooling with EGIN

Dataset	BACE	BBBP	MUV	HIV	SIDER	Tox21	Clintox
**Molecules**	1513	2039	93 087	41127	1402	7815	1476
**Graph size***	97	132	46	222	492	132	136
**Metric**	AUC-ROC (higher is better)						
**DiffPool (EGIN)**	0.797 +/− 0.054	0.842 +/− 0.054	0.711 +/− 0.033	0.722 +/− 0.024	–	0.777 +/− 0.023	0.893 +/− 0.074
**MinCut (EGIN)**	0.805 +/− 0.054	0.822 +/− 0.064	0.695 +/− 0.050	0.721 +/− 0.025	–	–	0.890 +/− 0.076
**Metric**	AUC-PRC (higher is better)						
**DiffPool (EGIN)**	0.777 +/− 0.064	0.934 +/− 0.039	0.029 +/− 0.015	0.213 +/− 0.055	–	0.365 +/− 0.044	0.816 +/− 0.099
**MinCut (EGIN)**	0.788 +/− 0.069	0.923 +/− 0.043	0.037 +/− 0.020	0.224 +/− 0.048	–	–	0.806 +/− 0.104

Note: **Graph size*** is the number of nodes of the largest molecule in the dataset; ‘-’ means failed to obtain a valid result.

### Hyperparameters

We list the optimized hyperparameters of different models here in Table 10.

**Table 9:** Regression benchmark results of dense pooling with EGIN

Dataset	ESOL	FreeSolv	Lipophilicity
**Molecules**	1128	639	4200
**Graph size***	155	24	115
**Metric**	RMSE (lower is better)		
**DiffPool (EGIN)**	1.314 +/− 0.322	2.998 +/− 0.630	0.859 +/− 0.110
**MinCut (EGIN)**	1.342 +/− 0.417	–	0.823 +/− 0.064

Note: **Graph size*** is the number of nodes of the largest molecule in the dataset; ‘-’ means failed to obtain a valid result.

**Table 10:** Optimized hyperparameters

Hyperparameter	Hidden dims	Dropout rate	Learning rate	Pooling ratio
**Classification**				
**GCN**	64	0.6	0.1	–
**GAT**	128	0.4	0.001	–
**GIN**	256	0.4	0.01	–
**GraphSAGE**	128	0.4	0.01	–
**TopKPool (GIN)**	64	0.4	0.01	0.7
**SAGPool (GIN)**	64	0.4	0.001	0.5
**EdgePool (GIN)**	128	0.4	0.001	–
**ASAP (GIN)**	256	0.6	0.001	0.7
**DiffPool (GIN)**	128	0.2	0.001	0.5
**MinCut (GIN)**	128	0.2	0.001	0.5
**TopKPool (EGIN)**	64	0.6	0.01	0.7
**SAGPool (EGIN)**	64	0.4	0.01	0.7
**EdgePool (EGIN)**	256	0.2	0.001	–
**DiffPool (EGIN)**	128	0.2	0.001	0.5
**MinCut (EGIN)**	128	0.6	0.001	0.7
**EGIN**	128	0.4	0.001	–
**MESPool (EGIN)**	128	0.2	0.001	0.5*
**Regression**				
**GCN**	128	0.6	0.001	–
**GAT**	128	0.2	0.001	–
**GIN**	256	0.2	0.01	–
**GraphSAGE**	128	0.4	0.001	–
**TopKPool (GIN)**	128	0.2	0.01	0.7
**SAGPool (GIN)**	128	0.2	0.01	0.7
**EdgePool (GIN)**	64	0.6	0.01	–
**ASAP (GIN)**	64	0.4	0.01	0.7
**DiffPool (GIN)**	64	0.2	0.001	0.3
**MinCut (GIN)**	64	0.2	0.01	0.5
**TopKPool (EGIN)**	128	0.4	0.01	0.7
**SAGPool (EGIN)**	128	0.2	0.001	0.7
**EdgePool (EGIN)**	64	0.4	0.01	–
**DiffPool (EGIN)**	128	0.2	0.001	0.3
**MinCut (EGIN)**	128	0.2	0.001	0.3
**EGIN**	64	0.4	0.001	–
**MESPool (EGIN)**	64	0.2	0.001	0.5*

Note: * The pooling ratio of MESPool denotes the threshold value.

### Dense pooling with edge features

EGIN based framework requires pooling layers to be able to update edge features in the pooling process; however, dense pooling methods initially lacked a reliable edge feature update operator. On the other hand, dense pooling process framework utilized mini-batches with a distinct data mode from the commonly employed GNN framework. For dense pooling, the node feature $X$ and the adjacency matrix $A$ has the shape [batch, nodes, n_feat] and [batch, nodes, nodes], respectively, while the edge feature $E$ has shape [batch, nodes, nodes, e_feat], in which the attributes of non-existing edges are all zeros. This makes the EGIN operation in dense mode not completely consistent with the standard one (such as the edge normalization), thereby making it hard to conduct experiments that are entirely fair.

In order to conduct experiments for dense pooling with EGIN, we update the edge feature in dense pooling by the following function:

\begin{align*}& E_{bike}^{\prime}=\sum_{j}\sum_{l}{{S^{T}}_{bij}E_{bjle}}S_{blk} \end{align*}

As we mentioned above, dense pooling requires much more computational resources than sparse pooling on large graphs, for the size of $A$, $X$, $E$ and $S$ is depend on the largest graph in the dataset. It is evident that this edge feature update operation will worsen the issue, leading to a substantial increase in both computation time and space. In our test, dense pooling with edge feature became the slowest methods. Additionally, when working with datasets that contain large-sized molecules (SIDER), dense pooling have an excessive GPU memory usage and failed to produce effective results within a reasonable time frame.

These issues significantly diminish the reference value of the benchmark results. Therefore, we present the benchmark results of dense pooling with EGIN here in Tables 8 and 9.
